# Risk-Assessment-Based Optimization Favours the Development of Albumin Nanoparticles with Proper Characteristics Prior to Drug Loading

**DOI:** 10.3390/pharmaceutics14102036

**Published:** 2022-09-24

**Authors:** Gábor Katona, Bence Sipos, Ildikó Csóka

**Affiliations:** Faculty of Pharmacy, Institute of Pharmaceutical Technology and Regulatory Affairs, University of Szeged, Eötvös Str. 6, H-6720 Szeged, Hungary

**Keywords:** nanomedicine, Quality by Design, albumin nanoparticle, quality control, factorial design

## Abstract

Albumin nanocarrier research and development is a challenging area in the field of personalized medicine and in providing advanced therapeutic solutions. Albumin as a biocompatible, nonimmunogenic, and non-toxic protein carrier that can be exploited to conjugate drugs with poor bioavailability to improve on this feature. With many different perspectives and desired target profiles, a systematic structural approach must be used in nanoparticle development. The extended Research and Development (R&D) Quality by Design thinking and methodology proved to be useful in case of specific nanoparticle development processes before. However, the coacervation method is the most frequently applied preparation method for HSA nanoparticles; there is a lack of existing research work which has directly determined the influence of process parameters, control strategy, or design space. With a quality-management-driven strategy, a knowledge space was developed for these versatile nanoparticles and an initial risk assessment was conducted on the quality-affecting factors regarding the coacervation method, followed by an optimization process via Plackett–Burman and Box–Behnken experimental design. As a result of screening the effect of process variables on the fabrication of HSA nanoparticles, an optimized colloidal drug delivery system was engineered with desired nanoparticulate properties.

## 1. Introduction

Meeting the needs of today’s pharmaceutical industry cannot be achieved without structured, time- and energy-efficient, thoroughly considered research and development processes, in addition to serving patients’ therapeutic expectations [1]. Current stakeholders’ demands and the utilization of innovative techniques lie in the field of nanomedicine, of which territory the current manufacturing processes require systematic and holistic approaches in order to develop a stable, long-lasting, reproducible nanomedical product on top of satisfying the production line [2,3]. This is especially true for protein-based drug carrier systems, in which case we are able to develop these systems capable of administering small- and large-molecule active substances achieving improved bioavailability. Solutions that emphasize mathematical modelling and quality improvement techniques based on risk assessment can provide answers to the problems arisen from industrial scale-up and manufacturing difficulties. This problem is particularly evident in the development of albumin-based carrier systems, where, due to high operation temperature (>60 °C) or high shear stress of albumin that tends to irreversible, many industrially adaptable productions methods fall out of the possible range where a nanomedical product with a qualitatively appropriate product profile can be developed [4].

However, with the application of appropriate development technologies, albumin-based carrier systems can be characterized by a number of beneficial properties that are capable of meeting therapeutic needs with improved treatment strategies that traditional carriers cannot or only to a small extent can meet [5,6]. Most specifically, human serum albumin (HSA) is used to fulfil the requirements of an advanced therapeutic system as it is endowed by high biocompatibility, biodegradability, and safety profile (reduced immunogenicity). It is less opsonized by the reticuloendothelial system (RES) through an aqueous steric barrier, which may increase the circulation half-life of the albumin-bound active substance [7]. HSA offers a high drug-loading capacity due to its multiple binding sites, where both lipophilic and hydrophilic drugs, regardless of their surface charge conditions, can bind to albumin efficiently. This is possible by the well-defined primary structure and high measure of charged amino acids (e.g., lysine) capable of forming electrostatic interactions with positively and negatively charged molecules [8]. This chemical-conjugation-driven nanoparticle formation assures that the active substance is protected from metabolic and elimination mechanisms and increases its drug release and permeability across crudely accessible and permeable biological barriers. HSA itself has a high tendency to provide active targeting without using external ligands as it has high affinity to the gp60 receptors, which are expressed on the endothelial cells, as well as to neonatal Fc receptor (FcRn), which is expressed in the intestines to a great extent [9]. The high affinity can be observed to secreted protein, acidic and rich in cysteine (SPARC) pathways as well as being responsible for regulation of cell growth [8,10].

In recent years, alternative drug delivery routes became the focus of research and development with the aim of satisfying patient expectations by applying simple and effective drug therapy avoiding invasive medication (e.g., parenteral administration) [11]. Utilization of alternative drug delivery routes of proteins, such as oral, nasal, or ocular routes, indicates several advantages (e.g., targeted therapy, bypassing biological barriers, a chance to minimize therapeutic dose, or minimization of side effects); however, from technological point of view it might be challenging. Nasal delivery of HSA already represents a significant impact in brain targeting with the aim to treat neurodegenerative diseases [12,13,14]. Proteins, as HSA, are commonly sensitive against the biological milieu; they can undergo irreversible structural changes mainly due to enzymatic degradation, and therefore the optimization of adequate formulation is required to exploit these special pathways [15].

To improve product stability and to control the drug release from the nanocarrier system on these administration routes, crosslinking is an essential step for HSA nanoparticle formulation. Stability-enhancing techniques and excipients provide opportunities that have a direct impact on the ability of the carriers to enclose the active substance and their effect in biological media [16]. Glutaraldehyde is the most commonly applied crosslinker for stabilizing HSA nanoparticles; however, due to its neurotoxicity and unfavourable reaction with the encapsulated drugs (such as mediating fast degradation and instant burst-like drug release prior to reaching the target biological compartment) it is advised to be avoided [17]. As an alternative, 1-ethyl-3-(3-dymethylaminpropyl) carbodiimide hydrochloride (EDC) is a suitable compound which is widely used due to its low cytotoxicity. EDC reacts with the biopolymers’ hydroxyl groups in order to form active O-urea, which creates an amide link with amino groups and releases the water soluble, easily removable isourea [18].

Optimization of nanocarriers prior to drug loading plays a pivotal role in achieving the predetermined goals of the formulation. This optimization strategy can be observed in polymeric micelles, where the blank micelles with the highest capacity of drug-loading and appropriate colloid chemical characteristics are chosen for further assessment [19]. The same can be claimed in the case of liposomes, where the selection of the optimal lipid composition precedes the drug loading [20]. Currently, the literature of albumin optimization techniques scarcely touches this subject, let alone yet in a quality-controlled environment. The risk-assessment- and factorial-design-based Quality by Design (QbD) methodology can be utilized to perform the initial drug carrier development to its full extent [21]. Followed by the proper establishment of knowledge space and determination of critical quality attributes (CQAs), the critical material attributes (CMAs) and critical process parameters (CPPs) of a well-structured, multi-level optimization design can be evaluated. The main cornerstones of the QbD methodology are adequate quality, safety, and therapeutic efficacy. Risk-assessment-based quality management provides a better understanding of the process parameters and the factors affecting the product quality. This quality management approach is described in specified guidelines of the International Council of Harmonisation of Technical Requirements for Pharmaceuticals for Human Use (ICH) [22,23,24]. Screening for optimizable factors can be performed via various methods. In multicomponent systems, it is common to utilize phase diagrams as tools to optimize the composition [25]. Another method which we applied is the evaluation of multi-level factorial design studies. The most common approach is to apply Plackett-Burman screening design, which has the benefit of finding the highly influencing factors with a decreased amount of trial runs. As an equivalent to the phase diagram methodology, it provides in-depth mathematical modelling. By exploiting this screening technology, further optimization can be more precise and accurate and it also minimizes the trials runs later, for instance, in a Box–Behnken-type or the classic 2^x^ factorial designs [26].

This present study lies on the hypothesis that *prior-to-drug-loading optimization* of albumin nanoparticles (i) increases the potential of the formation of drug-bound albumin nanoparticles with proper nanocharacteristics and (ii) provides nanoparticles with high drug-loading and encapsulation capacity. This approach is novel in the case of HSA nanoparticles, where the quality assurance was based on a structured and precisely defined target product profile. The methodology is applied to the widely applied, simple, and robust rapid coacervation method. To support our claims and accomplish the goals, an initial risk assessment was performed followed by a factorial-design-conducted optimization and a detailed colloidal characterization and statistical evaluation of the engineered has nanoparticles.

## 2. Materials and Methods

### 2.1. Chemicals and Reagents

Human serum albumin (HSA) (lyophilized powder, purity > 97%), 1-(3-dimethylaminopropyl)-3-ethylcarbodiimide hydrochloride (EDC), physiological saline solution, and sodium hydroxide (NaOH) to prepare 0.1 M NaOH solution were purchased from Sigma Aldrich Co. Ltd. (Budapest, Hungary). Analytical-grade ethanol (EtOH) was obtained from Merck KGaA (Darmstadt, Germany). In all experiments, water was purified by the Millipore Milli-Q^®^ Gradient Water Purification System.

### 2.2. Definition of the Quality Target Product Profile (QTPP)

Defining the target profile of the desired nanomedical product is of paramount importance and it is the first essential step in the QbD-based risk assessment process. The Quality Target Product Profile (QTPP) refers to all the requirements set up by technological solutions, the characteristics of the nanoparticle, and the desired pharmaceutical effect after administration. The definition of the QTPP was performed accordingly to the specified ICH guidelines [22,23].

### 2.3. Determination of CQA, CMA, and CPP Elements

CQAs are physicochemical, biological, or microbiological characteristics of the desired product which must meet the requirements of the goals set up as QTPPs to ensure appropriate product quality, safety, and efficacy. CPPs are related to the production method as well as CMAs, which production method in our case was the rapid coacervation method. Data and the selection of factors were based on a collection process via literature screening for albumin nanoparticle development and prior knowledge and experience.

### 2.4. Initial Risk Assessment

QbD is a risk-assessment (RA)-oriented approach where qualitative and quantitative risk factors expressed as severity scores must be presented. Risk assessment was performed in a two-step rating system using LeanQbD^®^ Software (QbD Works LLC, Fremont, CA, USA). Firstly, an interdependence rating was performed amongst the feasible QTPP and CQA elements and amongst CQA and CMA/CPP elements. A three-level scale was used to describe the relation between the parameters where “high” (H), “medium” (M), or “low” (L) attributes were assigned to each other. The assignment had the general quality control aspects: occurrence of the risk, controllability, eliminability, fixability, and detectability. To quantify these qualitative assigned relations, severity scores were calculated using the software. As a result, Pareto diagrams were generated, presenting the numeric severity score, and rankings of CQAs and CPPs were established [2,21].

### 2.5. Screening Study Using Plackett–Burman Design

At first, Plackett–Burman design was set up in order to investigate the effect on nanoparticle characteristics of 7 high-risk factors obtained from the RA procedure. As dependent variables, the average hydrodynamic diameter (Z-average) (Y_1_), polydispersity index (PdI) (Y_2_), and zeta potential (Y_3_) of the HSA nanoparticles were selected. Each independent factor was evaluated at low (−1) and high (+1) levels (Table 1). The determination of the low and high values was based on preliminary experiments. An 8-run factorial design was performed in triplicate. TIBCO Statistica^®^ 13.4 (Statsoft Hungary, Budapest, Hungary) software was used to generate and randomize a design matrix for statistical analysis. The relationship of the variables on the response was analysed by the following general equation:

(1) $$Y=\beta_{0}+\beta_{1}x_{1}+\beta_{2}x_{2}+\beta_{3}x_{3}+\beta_{4}x_{4}+\beta_{5}x_{5}+\beta_{6}x_{6}+\beta_{7}x_{7}$$

where *β*_0_ is a constant, *β*_1–7_ are linear coefficients, and *x*_1–7_ are the main effect factors. Analysis of variance (ANOVA) was performed to test the significance of the model and the factor coefficients. Differences were considered significant when the *p*-value was less than 0.05. Data is presented as mean ± SD.

### 2.6. Preparation of HSA Nanoparticles

**Figure 1:**
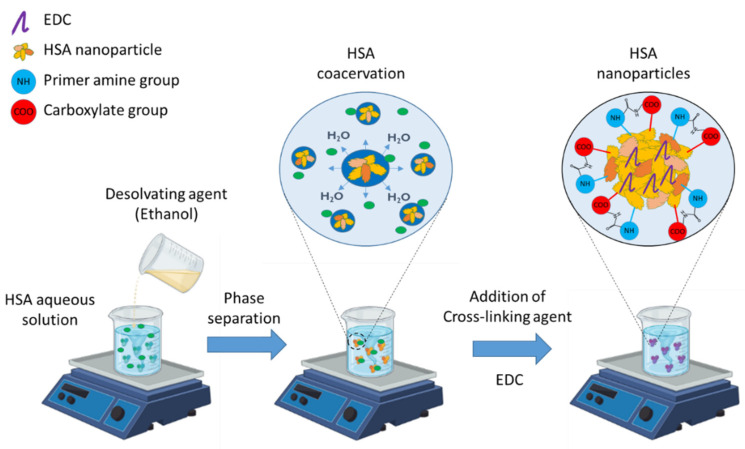
Preparation of HSA nanoparticles.

HSA nanoparticles were prepared by a rapid coacervation method (Figure 1) [27]. Briefly, HSA was dissolved in 2 mL of purified water or physiological saline solution with the set pH of 8.0 using 0.1 M NaOH solution. Then, nanoparticles were precipitated by the continuous addition of the coacervating agent ethanol to the HSA solution at a defined flow rate using a peristaltic pump under constant stirring using a magnetic stirrer (AREC.X heating magnetic stirrer, Velp Scientifica Srl, Usmate Velate, Italy) at a set ambient temperature of 25 °C. After finishing the coacervation process, determined volume of freshly prepared EDC aqueous solution (10 mg/mL) was added to induce crosslinking of free carboxyl and amino groups. The crosslinking process was continued under constant stirring of the nanoparticles for the predetermined incubation time. Followed by the incubation, the nanoparticles were purified by 30 min centrifugation at 4 °C and 16,500 rpm (22,413 RCF) in a Hermle Z323 laboratory centrifuge (Hermle AG, Gossheim, Germany) in two cycles, during which the pellet was redispersed in 1.5 mL of purified water using a vortex mixer (Biobase MX-S, Jinan, Shandong, China).

### 2.7. Optimizing the Rapid Coacervation Method for HSA Nanoparticle Formation

The rapid coacervation method applied to produce HSA nanoparticles was optimized using Box–Behnken factorial design. As independent variables, the investigated parameters with the highest significance determined by the Plackett–Burman screening design, i.e., the amount of ethanol, the concentration of HSA, and the incubation time, were considered in the optimization process. As potentially critical parameters influencing the Z-average (Y_1_), PdI (Y_2_), and zeta potential (Y_3_), the effect of these independent variables, as well as nanoparticle yield (Y_4_), for supporting the selection of optimal pH for preparation was investigated at 3 levels (−1; 0 and +1) in a 15-run trial (Table 2). The terminal factor values were directly adopted from the prior screening design. To construct the second-order polynomial models and to investigate the quadratic response surface of the trial, TIBCO Statistica^®^ 13.4 (Statsoft Hungary, Hungary) was applied. The relationship of the variables on the response can be analysed by the following second-order equation:

(2) $$Y=\beta_{0}+\beta_{1}x_{1}+\beta_{11}x_{1}^{2}+\beta_{2}x_{2}+\beta_{22}x_{2}^{2}+\beta_{3}x_{3}+\beta_{33}x_{3}^{2}$$

where *Y* is the response variable; *β*_0_ is a constant; *β*_1_, *β*_2_, and *β*_3_ are linear coefficients; *β*_11_, *β*_22_, and *β*_33_ are quadratic coefficients; *x*_1–3_ are the main effect factors; and *x*_1_^2^, *x*_2_^2^, and *x*_3_^2^ are the quadratic effect factors. Response surface plots were plotted in order to get a better understanding of the main effects and the interaction amongst the investigated factors, taking into account the change in the direction of the values of the examined dependent factors depending on the setting of the independent variables. The 3D response surface plots were plotted according to the regression model by keeping one variable at the centre level. The significance of the variables and interactions were evaluated using analysis of variance (ANOVA). Differences were considered significant when the *p*-value was less than 0.05. All experimental runs (formulations) were prepared in triplicate. Data is presented as mean ± SD.

### 2.8. Average Hydrodynamic Diameter, Polydispersity Index and Zeta Potential Determination

The Z-average, PdI, and zeta potential of HSA nanoparticles were determined by dynamic light scattering (DLS) in folded capillary cells using a Zetasizer Nano ZS (Malvern Instruments, Malvern, UK) at 25 °C. The refractive index of nanoparticles was 1.334. All measurements were repeated three times, and the average values of each were used for statistical evaluation in the selected optimization designs.

### 2.9. Determination a Nanoparticle Yield after Coacervation

For the determination of the percentage yield, HSA nanoparticles were separated from free albumin by centrifugation at 16,500 rpm (22,413 RCF) for 20 min at 4 °C in a Hermle Z323 laboratory centrifuge (Hermle AG, Gossheim, Germany). Aliquots of the supernatant were diluted 10-fold with purified water and the amount of free HSA in the supernatant was determined using a standard BCA protein assay. Briefly, 1000 µL of BCA working reagent was added to 50 µL of withdrawn supernatant and incubated for 30 min at 37 °C. Then, the samples were analysed spectrophotometrically at 562 nm. The free HSA content of the samples was determined based on the linear regression of the calibration curve. The calibration was performed using HSA standard solution in the 25 to 1000 µg/mL concentration range, where the determined coefficient of linearity (R^2^) value was 0.9982.

### 2.10. Raman Spectroscopy

Raman spectra of HSA nanoparticles at the key steps of preparation was performed using a Thermo Fisher DXR Dispersive Raman instrument (Thermo Fisher Scientific Inc., Waltham, MA, USA) equipped with a CCD camera and a diode laser operating at a wavelength of 780 nm, applying a laser power of 12 mW at a 50 µm slit aperture size. Each spectrum was recorded with an exposure time of 2 s and an acquisition time of 6 s, for a total of 32 scans per spectrum in the spectral range 3300–200 cm^−1^ with cosmic ray and fluorescence corrections.

## 3. Results

### 3.1. Determination of Quality Target Product Profiles

QTPPs were considered regarding the general nanoparticle characteristics of the formulation. The chosen elements contain factors with specific colloid chemical and biopharmaceutical aspects that strongly influence the quality and usability of the product for medicinal purposes (Table 3).

### 3.2. Initial Risk Assessment on the Rapid Coacervation Method for HSA Nanoparticle Development

**Figure 2:**
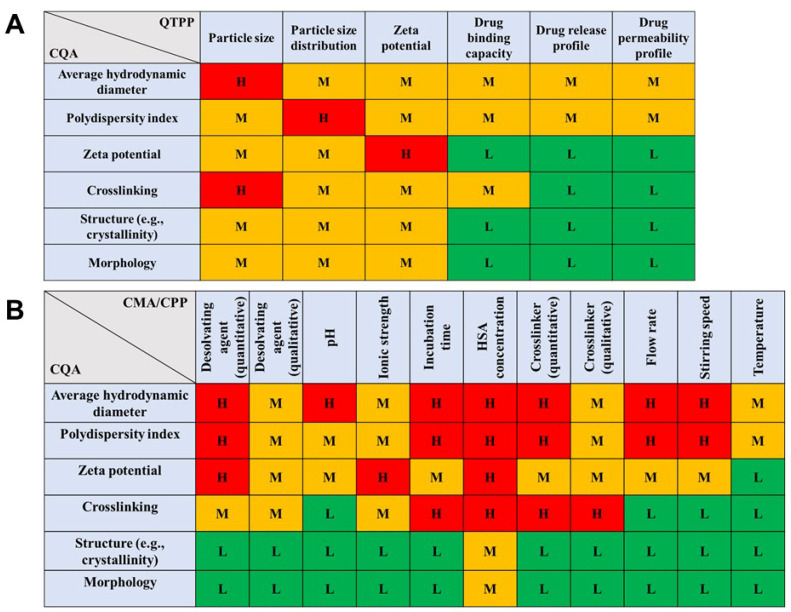
Interdependence rating amongst CQA–QTPP (**A**) and CPP/CMA–CQA (**B**) elements. The relations between the elements were assigned with “H”—high, “M”—medium, and “L”—low attributives.

The risk assessment was performed as stated before: first, the impact on the selected CQA elements on QTPPs was evaluated, followed by investigation amongst the CPP/CMA and CQA elements (Figure 2). The design space was constructed based on an extensive literature review and preliminary experience. The coacervation method is the most commonly applied method to prepare HSA nanoparticles, during which the precipitation of nanoparticles is induced by the continuous addition of a water-miscible desolvating agent (e.g., methanol, ethanol, acetone) under constant stirring.

The type and amount of desolvating agent are effective parameters with regards to the Z-average of HSA nanoparticles. Strop et al. studied the effect of solvents with different dielectric constants (µ) (µ_acetone_ < µ_ethanol_ < µ_methanol_) on the size of HSA nanoparticles. They concluded that desolvating agents with higher dielectric constants led to a smaller particle size, which can be explained with the slower decrease in the polarity of mixture, and therefore slower dehydration of protein [28]. In contrast, Mohammad-Beigi et al. obtained not so clear a correlation between the dielectric constant and Z-average, as well as no significant effect of different desolvating agents on the zeta potential. Based on their results, the use of ethanol can result in nanoparticles with the desired particle size (~150 nm) [29], which fits our criterion. The use of methanol and acetone was rejected because of safety issues. As methanol belongs to Class 2 solvents with the maximum permitted daily contamination of 3000 ppm [30], its residual in the final product can be extremely dangerous. The use of acetone could also be a rational choice; however, its effect on the unfolding of HSA is less remarkable than that of ethanol. Unfolding of albumin exposes its interactive sites, such as thiol and amine groups or other hydrophilic regions, to relocate to the surface, which makes a molecule capable of further intra-molecular binding, therefore increasing the potential to entrap bioactive compounds due to the reduction in hydrophobic interactions. Ethanol led to extreme changes in the content of the secondary structure of albumin, supporting its efficiency. The size and surface properties of has nanoparticles can be significantly changed by the number of disulphide bonds and thiol groups, degree of unfolding, electrostatic repulsion between protein molecules, pH, and ionic strength [31].

The flow rate and the volume of the added ethanol is also an important parameter to obtain a favourable size of albumin nanoparticles. Manual addition of desolvating agent by a syringe was already reported; however, using a peristaltic pump is more effective for carefully controlling the flow rate of desolvation agent addition [32]. Jahanban-Esfahlan et al. concluded that the addition of ethanol in an equal amount with the HSA solution led to fabricating nanoparticles with around a 100 nm Z-average [27]. Langer et al. reported that using 1.0 and 2.0 mL/min ethanol flow rates resulted in HSA nanoparticles with a more uniform particle size in the range between 100 and 200 nm and a narrow PdI [33]. However, stirring speed or temperature did not prove to be significant parameters on the particle size of albumin nanoparticles [34,35].

Langer et al. also revealed that, in the pH range of 7.5–9.0, the particle size decreased from 200 to 150 nm, but particle yield also decreased from 90 to 66%. Therefore, to find the golden mean of both important parameters, pH 8 was selected for formulation of HSA nanoparticles, where the particle yield was still 80% [33]. Sebak et al. supported our decision, according to which at higher pH (8.0 to 8.5) also leads to repulsion among the HSA molecules due to a steric effect, reducing particle size and avoiding aggregation of particles [36]. Ionic strength also showed a remarkable effect on the desolvation process; nanoparticles prepared in 10 mM NaCl solution showed a significantly higher Z-average in comparison to purified water at the same pH. The amount of HSA in the concentration range between 25 and 100 mg/mL indicated only a slight influence on the Z-average, with a shallow size minimum of 155 nm at 50 mg/mL HSA. Increasing the HSA concentration slightly reduced the PdI [33]. As crosslinker, 3–5 mg EDC is sufficient to crosslink the applied amount of HSA instead of glutaraldehyde to avoid toxicity issues and to reduce the time of the preparation process from overnight to 3 h [27].

Designing nanoparticles with consideration of these factors is the basic step towards an efficient and clinically translatable nanoproduct.

**Figure 3:**
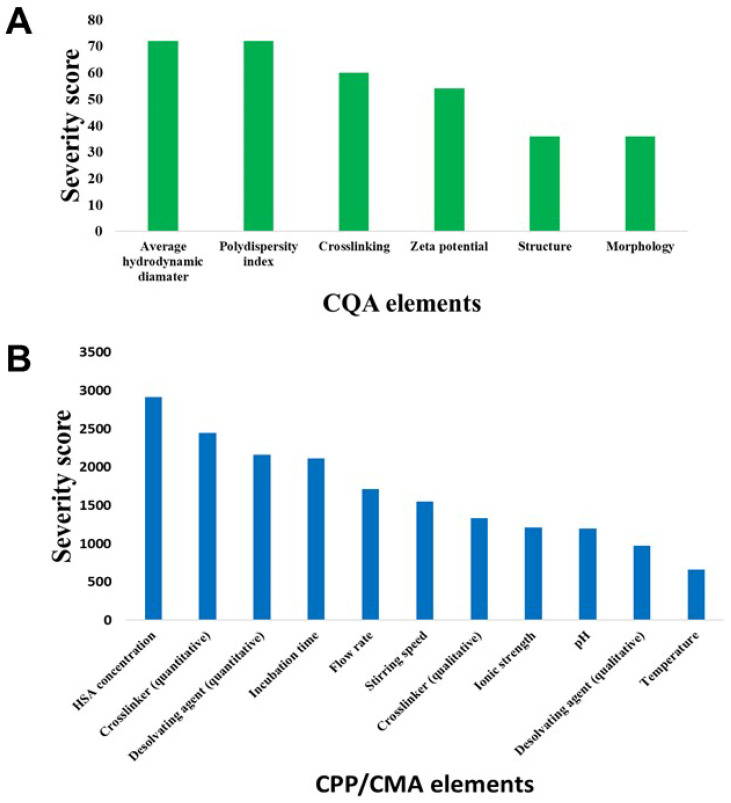
Probability-based quantitative estimation of possible risk factors associated with HSA nanoparticle formulation via the rapid coacervation preparation method. The software-calculated severity scores are depicted in the case of Critical Quality Attributes (**A**) and Critical Process Parameters/Critical Material Attributes (**B**).

To quantify the results of the knowledge-space-based interdependence rating, occurrence- and probability-based calculations were performed using the software engine and Pareto charts were generated (Figure 3).

Based on the risk assessment, seven high-risk factors were further evaluated in a Plackett–Burman screening factorial design. To cope with risk factors, two strategies can be exercised: either set a factor to a set point, or the opposite, vary them. In our case, the qualitative attributes of the crosslinker as EDC was set as a fix factor alongside a set pH of 8.0 and the usage of ethanol as desolvating agent, and all experiments were performed at ambient temperatures.

### 3.3. Screening of Optimizable Factors via Plackett–Burman Design

Plackett–Burman design was performed for screening the potential high-risk factors obtained from the risk assessment results in order to identify the most significant process variables affecting the CQAs and the desired nanoparticle characteristics. Plackett–Burman design is a two-level-based factorial screening design which can estimate the significant factors from numerous factors with very high efficiency and accuracy. Therefore, it is a rational choice for reducing the number of high-risk factors that need to be studied in further experiments. Different HSA nanoparticle formulations were prepared by varying the process variables, then the Z-average (Y_1_), PdI (Y_2_), and zeta potential (Y_3_) of the formulations were measured to determine the response of selected process variables (Table 4).

#### 3.3.1. Screening the Effect of Process Variables on Average Hydrodynamic Diameter

The Plackett–Burman Experimental Design results revealed that varying the preparation process variables has a remarkable effect on the Z-average, resulting in a wide range of particle sizes from the micro to nano meter size range. The relationship between process variables and the Z-average (Y_1_) can be described with the following equation:

(3) $$Y_{1}=360.140+211.383x_{1}-193.484x_{2}-194.906x_{3}-206.977x_{4}-201.950x_{5}+210.400x_{6}+248.760x_{7}$$

The regression coefficient (R^2^) and adjusted R^2^ of the surface plot were 0.79919 and 0.71134, respectively. The positive coefficients before the independent variables of the linear model indicate an unfavourable course of action regarding the increase in the Z-average, while the negative coefficients indicate a favourable effect on the Y_1_. We found that all the investigated process variables (*x*_1_–*x*_7_) had significant effects on the Z-average (Table 5).

The results demonstrated that decreasing X_1_ (amount of ethanol), X_6_ (stirring speed), and X_7_ (concentration of NaCl), while increasing X_2_ (amount of crosslinker), X_3_ (incubation time), X_4_ (concentration of HSA), and X_5_ (ethanol flow rate) would contribute to the reduction in the Z-average of HSA nanoparticles. As all factors showed different levels of significance, it can be claimed that the build-up of the knowledge space and the initial risk assessment were performed successfully and the quality-controlled environment was set up properly.

#### 3.3.2. Screening the Effect of Process Variables on the Polydispersity Index

Despite all seven of the high-risk investigated factors showing some level of significance on the Z-average, the same cannot be claimed concerning the polydispersity index of the albumin particles. The relationship between process variables and the PdI (Y_2_) can be described with the following equation:

(4) $$Y_{2}=0.516-0.035x_{1}-0.067x_{2}-0.052x_{3}+0.015x_{4}-0.146x_{5}+0.006x_{6}+0.099x_{7}$$

The regression coefficient (R^2^) and the adjusted R^2^ of the surface plot were obtained as 0.77536 and 0.67708, respectively. The effect of the process variables on the PdI is presented in Table 6. The positive coefficients before the independent variables of the linear model indicate in this case an unfavourable effect, while the negative coefficients indicate a favourable effect on the Y_2_. The results revealed that decreasing *x*_7_ (concentration of NaCl) while increasing *x*_2_ (amount of crosslinker) and *x*_5_ (ethanol flow rate) would result in the decrease in the PdI of HSA nanoparticles.

The highest level of significance (** *p* < 0.01) can be observed in case of the ethanol flow rate and the ionic strength of the formulation expressed as the concentration of sodium chloride. The high speed and high-volume addition of the desolvating agent showed that it leads to the decrease in the PdI. This can be due to the fact that, by slow addition of the agent, the potential of aggregation would increase as free, non-coacervated albumin particles can bond with the coacervated moiety. Therefore, in the case of rapid desolvation, uniform-sized particles can form. As an increase in salt concentration includes a risk potential for surface-charge-mediated aggregation, it is also an unbeneficial factor when increased. Data in the literature also support our claim, as most types of generally applied buffer solutions or salts can interfere with the desolvation process [27].

#### 3.3.3. Screening the Effect of Process Variables on Zeta Potential

The impact of process variables on the zeta potential was also investigated with Plackett–Burman Design. Based on the ANOVA analysis, most of the investigated variables have significant effects on the zeta potential. The relationship between process variables and the zeta potential (Y_3_) can be described with the following equation:

(5) $$Y_{3}=-12.92-2.94x_{1}-3.42x_{2}+0.78x_{3}-5.26x_{4}-1.43x_{5}-0.59x_{6}+3.75x_{7}$$

The regression coefficient (R^2^) and the adjusted R^2^ of the surface plot were obtained as 0.92344 and 0.88994, respectively. The impact of process variables on the zeta potential can be seen in Table 7.

The results demonstrated that increasing X_1_ (amount of ethanol), X_2_ (amount of cross linker), X_4_ (concentration of HSA), X_5_ (ethanol flow rate), and X_6_ (stirring speed) while decreasing X_7_ (concentration of NaCl) would contribute to the increase in colloidal stability of HSA nanoparticles. Comparing the three responses, it can be clearly seen that increasing X_2_ (amount of cross linker) and X_5_ (ethanol flow rate) results in a lower Z-average, a narrower PdI, and higher colloidal stability, while when increasing X_7_ (concentration of NaCl), the aggregation tendency will increase. A partially negative resultant effect was obtained, in the case of increasing X_1_ (amount of ethanol), on the Z-average; X_3_ (incubation time), on colloidal stability; X_4_ (concentration of HSA), on the PdI; and X_6_ (stirring speed), on both the Z-average and PdI. Therefore, for further optimization, the refinement of previously mentioned process variables is required.

### 3.4. Optimization of HSA Nanoparticles with Box–Behnken Experimental Design

For Box–Behnken experimental design, the first step was the careful selection of three most significant variables out of the seven based on the evaluation of Plackett–Burman screening design. For this purpose, the values of those variables were fixed, which indicated a clear response on the investigated nanoparticulate properties (Y_1_–Y_4_). The factor-screening results showed that the nanoparticle yield (Y_4_) of the different formulations of Box–Behnken design ranged from 78 to 98%, which was appropriate as the pH of the formulation was set to 8.0 [33,36]. As a result, the Y_4_ response was not included in the optimization process. Two of these fixed factors were X_2_ (amount of crosslinker—EDC) and X_5_ (ethanol flow rate), which were maximized to 5 mg and 2 mL/min, respectively. An ethanol flow rate of 2 mL/min is appropriate to result in fast desolvation of HSA, while 5 mg EDC amount ensures adequate crosslinking of nanoparticles in the present concentration, indicating a low Z-average, a narrow PdI, and increased colloidal stability. Another two fixed factors were X_6_ (stirring speed) and X_7_ (concentration of NaCl); however, their values were minimized to 750 rpm and 0 *w*/*v*%, respectively, to avoid an increased Z-average and PdI, while decreasing the zeta potential, i.e., colloidal stability. On an exclusion basis, three factors remained, X_1_, the amount of ethanol (mL) as coacervating agent; X_3_, the incubation time (h) for crosslinking of nanoparticles; and X_4_, the concentration of HSA (mg/mL), whose effect required a more complex statistical analysis. Therefore, the effect of these variables were further investigated in a three-factor, three-level Box–Behnken experimental design with a total run number of 15 (Table 8). The number of experiments included the replicated centre points. The specification of low and high levels of factors was based on the previous Plackett–Burman design, whereas the medium levels were set as the average value of the low and high levels.

#### 3.4.1. Influence of Process Variables on the Z-Average (Box–Behnken Design)

The influence of selected process variables on the Z-average of HSA nanoparticles was investigated using a Box–Behnken response surface methodology. A quadratic equation describing the individual main effects of *x*_1_, *x*_3_, and *x*_4_ on Y_1_ was generated:

(6) $$Y_{1}=245.950-146.900x_{1}-69.213x_{1}^{2}-7.225x_{2}-28.613x_{2}^{2}-31.575x_{3}-1.887x_{3}^{2}$$

The regression coefficient (R^2^) and the adjusted R^2^ of the surface plot were obtained as 0.94978 and 0.91211, respectively. The negative coefficients before the independent variables of the linear model indicate the favourable effect on Y_1_. The significance of the effect of investigated process variables (*x*_1_, *x*_3_, and *x*_4_) on the Z-average is presented in Table 9.

**Figure 4:**
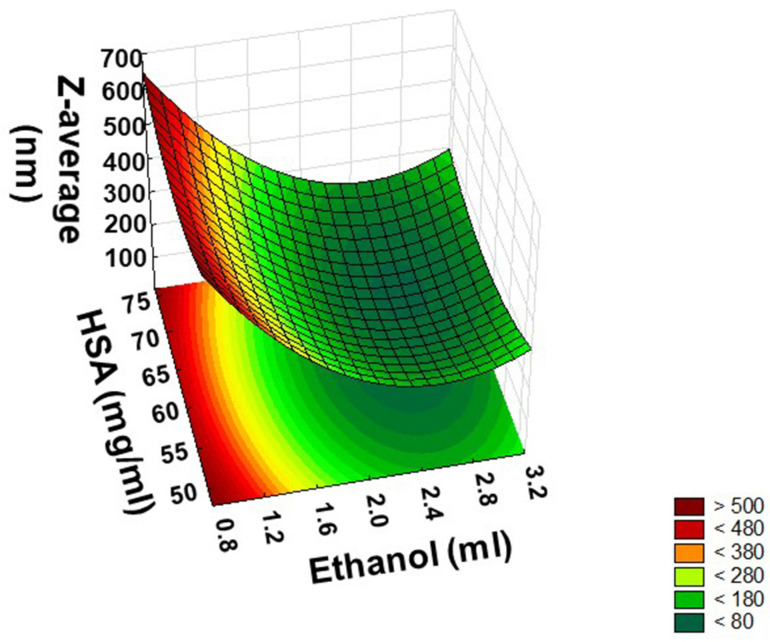
Response surface showing the effects of the amount of ethanol (X_1_) and the concentration of HSA (X_4_) at the mid-level of incubation time (X_3_) on the Z-average (Y_1_).

Based on the ANOVA analysis, it can be clearly seen that the amount of ethanol as coacervating agent was significant in terms of both the linear and quadratic effect on the Z-average. The concentration of HSA also showed a significant quadratic effect, while its linear effect was negligible. However, incubation time for crosslinking proved to be insignificant in the applied concentration range. For easier interpretation of the significant impact of variables, the surface plot of particle size may provide more information (Figure 4).

On the surface plot, a minimum point can be identified indicating that the levels of factors were selected in an appropriate range to find the optimal ratio of HSA and Ethanol, which fits the general criteria of an adequate particle size range. In fact, particle size is a key attribute for a successful drug delivery system, as it affects the drug-loading and release behaviour as well as particle pharmacokinetics, biodistribution, and biological fate. Nanoparticles with a lower Z-average have a larger surface-area-to-volume ratio and thus can encapsulate less drug; moreover, they release it at a faster rate, with the drug being closer to the surface. Based on data in the literature, the optimal particle size of HSA nanoparticles should be in the size range of 100–200 nm to ensure adequate drug release and to reduce the chance of uptake by macrophages after absorption [37]. A Z-average of <200 nm is also advantageous for passive targeting, especially to tumour tissue, by the EPR effect [38]. Moreover, the smaller the particle size of the nanoparticles, the larger the specific surface area and the higher the loading efficiency. Taking into account these facts, the applied amount of ethanol should be 2.5 mL, while the applied concentration of HSA should be 62.5 mg/mL to reach the desired Z-average.

#### 3.4.2. Influence of Process Variables on the PdI (Box–Behnken Design)

The impact of selected process variables (*x*_1_, *x*_3_, and *x*_4_) on the PdI (Y_2_) of HSA nanoparticles can be described with the following generated quadratic equation:

(7) $$Y_{2}=0.661-0.208x_{1}-0.129x_{1}^{2}+0.017x_{2}-0.023x_{2}^{2}-0.006x_{3}+0.010x_{3}^{2}$$

The regression coefficient (R^2^) and the adjusted R^2^ of the surface plot were obtained as 0.9338 and 0.84416, respectively. The positive coefficients before the independent variables of the linear model indicate, in this case, an unfavourable effect, while the negative coefficients indicate a favourable effect on Y_2_. The significance of the effect of investigated process variables (*x*_1_, *x*_3_, and *x*_4_) on the PdI is demonstrated in Table 10.

**Figure 5:**
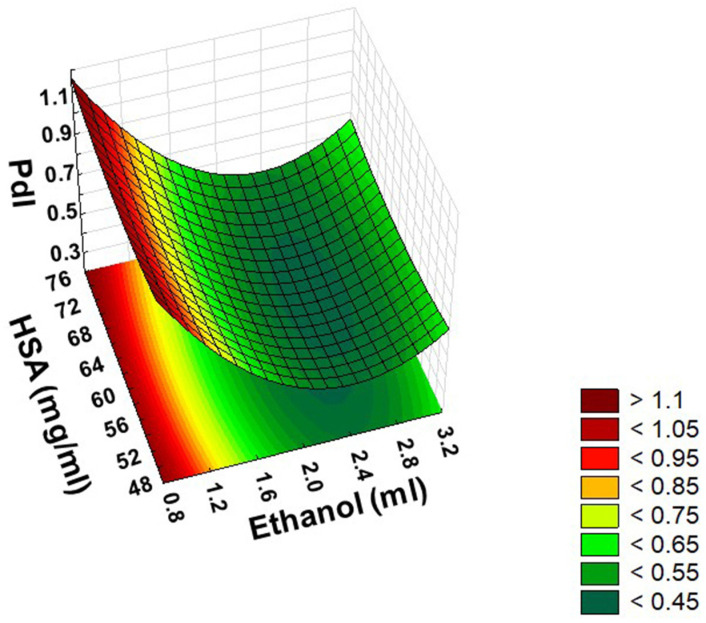
Response surface showing the effects of the amount of ethanol (X_1_) and the concentration of HSA (X_4_) at the mid-level of incubation time (X_3_) on the PdI (Y_2_).

The ANOVA analysis clearly demonstrated that the amount of ethanol as coacervating agent was significant in terms of both the linear and quadratic effect on PdI. However, incubation time for crosslinking and the concentration of HSA proved to be insignificant at the applied variable levels. For easier interpretation of the significant effect of the variables, the surface plot of the PdI can also provide more information (Figure 5).

The surface plot of the PdI has also a minimum point that claims the levels of factors were as well selected in an appropriate range to define the optimal ratio of HSA and ethanol in order to result in a monodisperse distribution of nanoparticles. Based on data in the literature, the narrower the PdI, the more colloidally stable the nanoparticulate system [39]. The PdI of HSA nanoparticles can be accepted in the range of 0.2–0.7, but it should be as small as possible [40].

#### 3.4.3. Influence of Process Variables on the Zeta Potential (Box–Behnken Design)

The influence of selected process variables *x*_1_, *x*_3_, and *x*_4_ on the zeta potential (Y_3_) of HSA nanoparticles was also examined. This relationship can be described with the following generated quadratic equation:

(8) $$Y_{3}=-5.62+0.14x_{1}+0.35x_{1}^{2}-1.08x_{2}-0.42x_{2}^{2}-1.28x_{3}+0.05x_{3}^{2}$$

Based on the R^2^ (0.53895) and adjusted R^2^ (0.19316) of the surface plot that was obtained, a poor correlation can be established, which was also supported with the ANOVA results (Table 11).

Based on the ANOVA analysis, all the investigated variables indicated an insignificant effect both in the linear and quadratic approach at the applied variable levels, which can be mostly claimed with the poor regression coefficients. Apart from an increase in the incubation time of the crosslinking reaction presuming increased colloidal stability through a linear effect, increasing the concentration of HSA presumes the same effect both linearly and quadratically. As the zeta potential is also a crucial nanoparticulate property, the variables which have a favourable effect on it should be taken into account as well at the optimization process. Particles aggregation via the bridging effect can occur in the case of HSA, due to its excessively high molecular weight and non-uniform surface [37]. According to the literature, the higher absolute value of the zeta potential influences the particle stability due to increased repulsion forces avoiding aggregation, cellular uptake, and intracellular trafficking [38].

### 3.5. Confirmation Test of the Model

**Figure 6:**
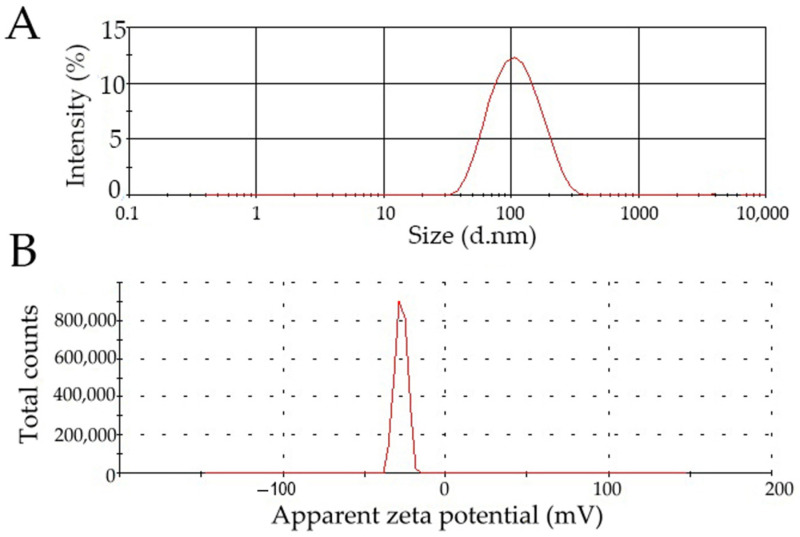
Particle size distribution (**A**) and zeta potential plot (**B**) of optimized HSA nanoparticles.

To confirm the model, HSA nanoparticles were fabricated according to the optimized values of process variables determined in the design of experiment (Table 12). Prepared nanoparticles were characterized, and the response was compared to the predicted value. The particle size distribution and zeta potential plot of optimized nanoparticles are presented in Figure 6.

The results indicate that the predicted and observed response values of the formulations with the optimized variables were nearly similar (within SD). A good agreement was obtained between the model prediction and experimental observation. Thus, the validity of the model was established.

### 3.6. Raman Spectroscopic Structural Investigation

**Figure 7:**
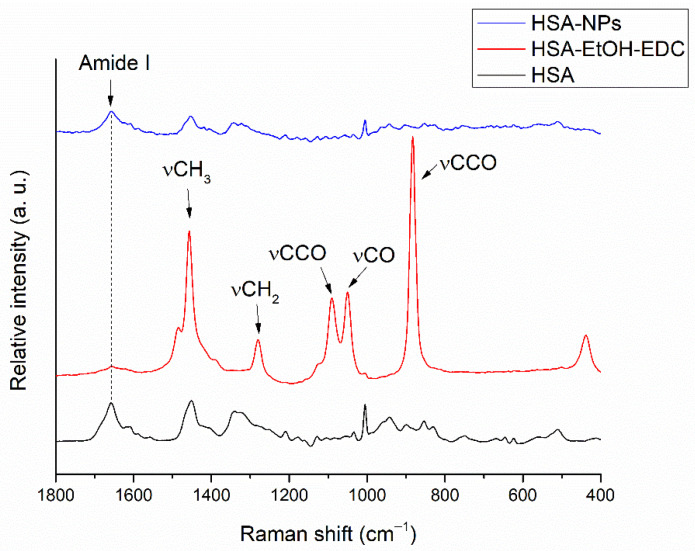
Raman spectrum of HSA aqueous solution (HSA), HSA nanoparticles after desolvating with EtOH and crosslinking with EDC (HSA-EtOH-EDC), and HSA nanoparticles after purifying with centrifugation (HSA-NPs).

Using ethanol at concentrations above 30% causes changes in the secondary structure of albumin by unfolding, indicating change in the α-helical structure. To investigate the reversibility of unfolding after purifying the nanoparticles, Raman spectroscopic studies were conducted. The secondary structure of HSA was studied at each key step of HSA nanoparticle preparation; Raman spectra are shown in Figure 7.

The most remarkable change for unfolding and refolding in the Raman spectrum can be observed at the peak centred at 1655 cm^−1^ in the amide I region, which is usually attributed to the ordered *α*-helix (*ho*). In the Raman spectrum of HSA-EtOH-EDC, the decrease in the fraction of the ordered *α*-helix (*ho*) indicates unfolding of HSA [41]. The appearing peaks at 888 cm^−1^ (CCO skeleton symmetric stretching vibration); 1054 cm^−1^ (CO scaling); 1104 cm^−1^ (CCO skeleton stretching vibration); 1287 cm^−1^ (CH2 deformation); and 1462 cm^−1^ (CH3 antisymmetric deformation) are unique to ethanol, the desolvating agent [42]. The absence of these characteristic peaks in the spectrum of HSA-NPs indicates a successful purification process, while the increased fraction of the ordered *α*-helix (*ho*) in comparison to HSA-EtOH-EDC supports partial refolding of HSA after redispersion of the nanoparticles in purified water due to hydrophobic interactions.

## 4. Discussion

This study focused on the risk-assessment-based development of HSA nanoparticles as a suitable colloidal carrier for targeted drug delivery. As an advanced drug delivery system, it has a significant role in future medicine. However, to achieve this, a sustainable, reproducible, and robust preparation method is required. Herein, a comprehensive QbD-based development approach is provided for the development of HSA nanoparticles, which can be generally used for designing a colloidal drug delivery system with desired nanoparticulate properties.

Several studies deal with the optimization of HSA nanoparticles; nevertheless, the exact value of some process parameters is often undefined. The rapid coacervation method is the simplest and most frequently applied preparation method, during which, due to the addition of an organic solvent (ethanol or acetone), the solubility of HSA decreases, resulting in precipitation into nanoparticles. However, the exact amount of required coacervating agent is not defined; in the most cases, it is suggested to add as much as needed to obtain a turbid colloidal solution, which is not a discrete determination for a reproducible process. Similar important process variables are, e.g., flow rate of the coacervating agent, concentration of HSA, amount of crosslinker, incubation time of the crosslinking reaction, pH, stirring speed, and ionic strength, which should be also defined for establishing the control strategy. For that purpose, QbD is an essential tool.

After the initial risk assessment, Plackett–Burman screening design was applied to identify the most significant process variables affecting the CQAs. Based on the regression analysis, the value of those variables was fixed, which indicated a clear response on the investigated nanoparticulate properties (Y_1_–Y_3_). Two of these fixed factors were X_2_ (amount of crosslinker—EDC) and X_5_ (ethanol flow rate), which were maximized to 5 mg and 2 mL/min, respectively, as these increased values indicated a low Z-average, a narrow PdI, and increased colloidal stability. Another two fixed factors were X_6_ (stirring speed) and X_7_ (concentration of NaCl); however, their values were minimized to 750 rpm and 0 *w*/*v*%, respectively, to avoid an increased Z-average and PdI, while decreasing the zeta potential i.e., colloidal stability.

The remaining three variables, X_1_, the amount of ethanol (mL) as coacervating agent; X_3_, the incubation time (h) for crosslinking of nanoparticles; and X_4_, the concentration of HSA (mg/mL), of which the effects were not totally clear, were further investigated in the Box–Behnken experimental design. It was revealed that the amount of ethanol as coacervating agent and the concentration of HSA had significant effects on the Z-average. However, incubation time for crosslinking proved to be insignificant in the applied concentration range. In terms of the PdI, the amount of ethanol as coacervating agent had a significant effect, but incubation time for crosslinking and the concentration of HSA proved to be insignificant at the applied variable levels. In the case of zeta potential, all the investigated variables were indicated as insignificant at the applied variable levels, which can be mostly claimed with the poor regression coefficients.

The optimization model of has nanoparticle preparation was confirmed by comparing the response of optimized process variables on nanoparticulate properties (Z-average, PdI, and zeta potential) to the predicted values. The results established the validity of the model as the predicted and the observed response values of the formulations with the optimized variables were nearly similar (within the SD). Based on the results, it can be claimed that a fundamental optimization strategy of HSA nanoparticles was established, which can be further utilized for the development of drug loading of HSA-based drug delivery systems.

## 5. Conclusions

In conclusion, this study demonstrated how the risk-assessment- and quality-control-driven QbD methodology can be utilized for the development of an HSA-based colloidal carrier which is suitable for improving drug delivery. With the fundamental understanding of the process variables affecting the coacervation method, the current case study provides a comprehensive development approach, which can be applied in general for designing HSA nanoparticles with desired nanoparticulate properties.

## Figures and Tables

**Table 1:** The 7 high-risk factors based on risk assessment and their lower and upper levels applied in the Plackett–Burman screening design in order to screen for optimizable factors regarding the rapid coacervation method to develop HSA nanoparticles.

Formulation Variables	Code	Levels	
		−1	+1
Amount of ethanol (mL)	X_1_	2	4
Amount of crosslinker (EDC) (mg)	X_2_	2.5	5
Incubation time (h)	X_3_	1	3
Concentration of HSA (mg/mL)	X_4_	50	75
Ethanol flow rate (mL/min)	X_5_	1	2
Stirring speed (rpm)	X_6_	750	1500
Concentration of NaCl (*w*/*v*%)	X_7_	0	0.9

**Table 2:** Independent variables selected for further optimization after the screening study, their codes, and the investigated values in the 3-level, 15-run Box–Behnken factorial design.

Independent Variables	Code	Levels		
		−1	0	+1
Amount of ethanol (mL)	X_1_	1	2	3
Incubation time (h)	X_3_	3	4.5	6
Concentration of HSA (mg/mL)	X_4_	50	62.5	75

**Table 3:** Selected Quality Target Product Profile (QTPPs) of HSA nanoparticles with the desired aim to optimize these particles prior to drug loading.

QTPP Element	Aim	Justification
Particle size (expressed as average hydrodynamic diameter)	<200 nm	Nanoparticles below 200 nm show increased surface area, which contributes to a highly diffusive area of drug release and permeation tendencies.
Particle size distribution (expressed as polydispersity index)	<0.300	PdI below 0.3 results in a uniform drug release and permeability profile, allowing controllable targeting and drug administration. Uniform particle size prior to drug loading also increases the potential of the formation of uniform drug-bound albumin particles.
Zeta potential	>│15 mV│	Generally speaking, nanoparticles with a zeta potential value above an absolute value of 15 mV (in either charge direction) are considered stable, as the repulsion forces are increased amongst particles.
Drug-binding capacity	Capable of binding the required amount of active substance	It depends on the target active substance concentration, the desired administration route, and the chosen dosage form. Generally, it should be as high as possible based on the reachable equilibrium amongst the active substance and albumin.
Drug release profile	Depends on the active substance, additional excipients, the administration route, and the expected pharmacokinetic profile.	
Drug permeability profile	Depends on the active substance, additional excipients, the administration route, and the expected pharmacokinetic profile.	

**Table 4:** Plackett–Burman screening design of experiments and their results: X_1_: Amount of EtOH (mL), X_2_: Amount of cross linker (EDC) (mg), X_3_: Incubation time (h), X_4_: Concentration of HSA (mg/mL), X_5_: Ethanol flow rate (mL/min), X_6_: Stirring speed (rpm), X_7_: Concentration of NaCl (*w*/*v*%), Y_1_: Z-average (nm), Y_2_: PdI, and Y_3_: zeta potential (mV). Data are presented as mean ± SD, (*n* = 3).

	Process Variables							Response		
Batch ID	X_1_ (mL)	X_2_ (mg)	X_3_ (h)	X_4_ (mg/mL)	X_5_ (mL/min)	X_6_ (rpm)	X_7_ (*w*/*v*%)	Y_1_ (nm)	Y_2_	Y_3_ (mV)
PB1	2	2.5	1	75	2	1500	0	90 ± 11	0.445 ± 0.065	−18.37 ± 4.65
PB2	4	2.5	1	50	1	1500	0.9	1828 ± 103	0.836 ± 0.044	−3.37 ± 2.94
PB3	2	5	1	50	2	750	0.9	194 ± 16	0.405 ± 0.169	−6.04 ± 0.88
PB4	4	5	1	75	1	750	0	109 ± 19	0.401 ± 0.106	−27.07 ± 3.96
PB5	2	2.5	3	75	1	750	0.9	181 ± 19	0.818 ± 0.052	−5.25 ± 2.06
PB6	4	2.5	3	50	2	750	0	116 ± 4	0.230 ± 0.033	−11.00 ± 0.26
PB7	2	5	3	50	1	1500	0	131 ± 25	0.430 ± 0.023	−10.27 ± 1.31
PB8	4	5	3	75	2	1500	0.9	233 ± 9	0.190 ± 0.086	−22.03 ± 3.45

**Table 5:** Effects of the 7 investigated processes and material parameters on the Z-average of HSA nanoparticles produced by the rapid coacervation method. Statistical analysis: One-way ANOVA, α = 0.05. (* *p* < 0.05; ** *p* < 0.01 significant).

Variable	Code	Significance	Effect of Variable on Z-Average
Amount of ethanol (mL)	X_1_	** *p* < 0.01	+
Amount of crosslinker (EDC) (mg)	X_2_	* *p* < 0.05	−
Incubation time (h)	X_3_	* *p* < 0.05	−
Concentration of HSA (mg/mL)	X_4_	** *p* < 0.01	−
Ethanol flow rate (mL/min)	X_5_	* *p* < 0.05	−
Stirring speed (rpm)	X_6_	** *p* < 0.01	+
Concentration of NaCl (*w*/*v*%)	X_7_	** *p* < 0.01	+

“+” indicates increasing the value of variable leads to the increase in Z-average, while “−” indicates increasing the value of variable leads to the decrease in Z-average.

**Table 6:** Effect of the 7 investigated process and material parameters on the PdI of HSA nanoparticles produced by the rapid coacervation method. Statistical analysis: One-way ANOVA, α = 0.05. (* *p* < 0.05; ** *p* < 0.01 significant, while n.s. means non-significant effect).

Variable	Code	Significance	Effect of Variable on PdI
Amount of ethanol (mL)	X_1_	n.s.	−
Amount of crosslinker (EDC) (mg)	X_2_	* *p* < 0.05	−
Incubation time (h)	X_3_	n.s.	−
Concentration of HSA (mg/mL)	X_4_	n.s.	+
Ethanol flow rate (mL/min)	X_5_	** *p* < 0.01	−
Stirring speed (rpm)	X_6_	n.s.	+
Concentration of NaCl (*w*/*v*%)	X_7_	** *p* < 0.01	+

“+” indicates increasing the value of variable leads to polydisperse distribution, while “−” indicates increasing the value of variable leads to monodisperse distribution.

**Table 7:** Effect of the 7 investigated process and material parameters on the zeta potential of HSA nanoparticles produced by the rapid coacervation method. Statistical analysis: One-way ANOVA, α = 0.05. (* *p* < 0.05; ** *p* < 0.01 significant, while n.s. means non-significant effect).

Variable	Code	Significance	Effect of Variable on Colloidal Stability
Amount of ethanol (mL)	X_1_	** *p* < 0.01	+
Amount of crosslinker (EDC) (mg)	X_2_	** *p* < 0.01	+
Incubation time (h)	X_3_	n.s.	−
Concentration of HSA (mg/mL)	X_4_	** *p* < 0.01	+
Ethanol flow rate (mL/min)	X_5_	* *p* < 0.05	+
Stirring speed (rpm)	X_6_	n.s.	+
Concentration of NaCl (*w*/*v*%)	X_7_	** *p* < 0.01	−

“+” indicates increasing the value of variable leads to increase in the absolute value of zeta potential, while “−” indicates increasing the value of variable leads to decrease in the absolute value of zeta potential.

**Table 8:** Box–Behnken experimental design and its results: X_1_: Amount of EtOH (mL), X_3_: Incubation time (h), X_4_: Concentration of HSA (mg/mL), Y_1_: Z-average (nm), Y_2_: PdI, Y_3_: zeta potential (mV), and Y_4_: Yield (%). Data are presented as mean ± SD, (*n* = 3).

	Process Variables			Response			
Batch ID	X_1_ (mL)	X_3_ (h)	X_4_ (mg/mL)	Y_1_ (nm)	Y_2_	Y_3_ (mV)	Y_4_ (%)
BB1	1	4.5	50	485 ± 26	0.901 ± 0.019	−28.9 ± 3.6	83.3 ± 3.4
BB2	3	4.5	50	117 ± 11	0.436 ± 0.181	−24.5 ± 4.9	94.8 ± 2.8
BB3	1	4.5	75	489 ± 22	0.876 ± 0.107	−30.5 ± 4.7	82.7 ± 4.1
BB4	3	4.5	75	144 ± 7	0.652 ± 0.112	−37 ± 3.1	98 ± 1.2
BB5	1	3	62.5	434 ± 6	0.871 ± 0.109	−26.7 ± 6.3	78.2 ± 3.9
BB6	3	3	62.5	168 ± 21	0.412 ± 0.156	−35.2 ± 4.5	95.6 ± 1.5
BB7	1	6	62.5	308 ± 26	0.731 ± 0.124	−30 ± 4.2	83.5 ± 2.8
BB8	3	6	62.5	111 ± 8	0.507 ± 0.161	−36.4 ± 4.4	91.3 ± 3.1
BB9	2	3	50	197 ± 21	0.453 ± 0.113	−29.4 ± 2.6	88 ± 2.9
BB10	2	3	75	186 ± 19	0.442 ± 0.062	−29.5 ± 6.5	94.4 ± 2
BB11	2	6	50	196 ± 20	0.438 ± 0.172	−30.1 ± 7.4	92.6 ± 3.7
BB12	2	6	75	117 ± 18	0.429 ± 0.185	−26.7 ± 2.7	92.9 ± 2.5
BB13	2	4.5	62.5	110 ± 5	0.251 ± 0.051	−29.6 ± 4.6	92 ± 1.7
BB14	2	4.5	62.5	110 ± 6	0.253 ± 0.059	−30.2 ± 4.1	93.9 ± 2.1
BB15	2	4.5	62.5	119 ± 4	0.247 ± 0.063	−30.4 ± 4.2	92.5 ± 2.2

**Table 9:** Effect of the selected process and material parameters on the Z-average of HSA nanoparticles produced by the rapid coacervation method. Statistical analysis: One-way ANOVA, α = 0.05. (* *p* < 0.05; ** *p* < 0.01 significant, while n.s. means non-significant effect).

Variable	Code	Significance	Effect of Variable on Z-Average
Amount of ethanol (mL)	X_1_	** *p* < 0.01	−
Amount of ethanol (mL)	X_1_^2^	* *p* < 0.05	−
Incubation time (h)	X_3_	n.s.	−
Incubation time (h)	X_3_^2^	n.s.	−
Concentration of HSA (mg/mL)	X_4_	n.s.	−
Concentration of HSA (mg/mL)	X_4_^2^	* *p* < 0.05	−

“−“ indicates increasing the value of variable leads to decrease in the absolute value of zeta potential.

**Table 10:** Effect of the selected process and material parameters on the PdI of HSA nanoparticles produced by the rapid coacervation method. Statistical analysis: One-way ANOVA, α = 0.05. (** *p* < 0.01 significant, while n.s. means non-significant effect).

Variable	Code	Significance	Effect of Variable on PdI
Amount of ethanol (mL)	X_1_	** *p* < 0.01	−
Amount of ethanol (mL)	X_1_^2^	** *p* < 0.01	−
Incubation time (h)	X_3_	n.s.	−
Incubation time (h)	X_3_^2^	n.s.	+
Concentration of HSA (mg/mL)	X_4_	n.s.	+
Concentration of HSA (mg/mL)	X_4_^2^	** *p* < 0.01	−

“+” indicates increasing the value of variable leads to polydisperse distribution, while “−” indicates increasing the value of variable leads to monodisperse distribution.

**Table 11:** Effect of the selected process and material parameters on the zeta potential of HSA nanoparticles produced by the rapid coacervation method. Statistical analysis: One-way ANOVA, α = 0.05. (n.s. means non-significant effect).

Variable	Code	Significance	Effect of Variable on Colloidal Stability
Amount of ethanol (mL)	X_1_	n.s.	−
Amount of ethanol (mL)	X_1_^2^	n.s.	−
Incubation time (h)	X_3_	n.s.	+
Incubation time (h)	X_3_^2^	n.s.	−
Concentration of HSA (mg/mL)	X_4_	n.s.	+
Concentration of HSA (mg/mL)	X_4_^2^	n.s.	+

“+” indicates increasing the value of variable leads to increase in the absolute value of zeta potential, while “−” indicates increasing the value of variable leads to decrease in the absolute value of zeta potential.

**Table 12:** Model confirmation of the design of the experiment: X_1_: Amount of EtOH (mL), X_2_: Amount of crosslinker (EDC) (mg), X_3_: Incubation time (h), X_4_: Concentration of HSA (mg/mL), X_5_: Ethanol flow rate (mL/min), X_6_: Stirring speed (rpm), X_7_: Concentration of NaCl (*w*/*v*%), Y_1_: Z-average (nm), Y_2_: PdI, Y_3_: zeta potential (mV) and Y_4_: Yield (%). Data are presented as mean ± SD, (*n* = 3).

	Process Variables							Response			
	X_1_ (mL)	X_2_ (mg)	X_3_ (h)	X_4_ (mg/mL)	X_5_ (mL/min)	X_6_ (rpm)	X_7_ (*w*/*v*%)	Y_1_ (nm)	Y_2_	Y_3_ (mV)	Y_4_ (%)
Predicted	2.5	2.5	3	62.5	2	750	0	122	0.231	−28.4	-
Experimental								119 ± 5	0.236 ± 0.051	−27.2 ± 2.6	96.3 ± 1.7

## Data Availability

The data presented in this study are available on request from the corresponding author.
